# Stacking sequence variations in vaterite resolved by precession electron diffraction tomography using a unified superspace model

**DOI:** 10.1038/s41598-019-45581-6

**Published:** 2019-06-24

**Authors:** Gwladys Steciuk, Lukáš Palatinus, Jan Rohlíček, Salim Ouhenia, Daniel Chateigner

**Affiliations:** 1 Institute of Physics of the Czech Academy of Sciences, Na Slovance 2, Prague, Czech Republic; 2 Laboratoire de Physique, Faculté des Sciences et Sciences de l’ingénieur, Béjaïa, 06200 Algeria; 3 CRISMAT, Normandie Université, ENSICAEN, UNICAEN, CNRS UMR6508, 6 Bd Maréchal Juin, F-14050 Caen Cedex 4, France

**Keywords:** Biomaterials, Nanoscale materials

## Abstract

As a metastable phase, vaterite is involved in the first step of crystallization of several carbonate-forming systems including the two stable polymorphs calcite and aragonite. Its complete structural determination would consequently shed important light to understand scaling formation and biomineralization processes. While vaterite’s hexagonal substructure (*a*_0_ ~ 4.1 Å and *c*_0_ ~ 8.5 Å) and the organization of the carbonate groups within a single layer is known, conflicting interpretations regarding the stacking sequence remain and preclude the complete understanding of the structure. To resolve the ambiguities, we performed precession electron diffraction tomography (PEDT) to collect single crystal data from 100 K to the ambient temperature. The structure was solved ab initio and described over all the temperature range using a unified modulated structure model in the superspace group *C*12/*c*1(*α*0*γ*)00 with *a* = *a*_0_ = 4.086(3) Å, *b* = $\sqrt{{\bf{3}}}$*a*_0_ = 7.089(9) Å, *c* = *c*_0_ = 8.439(9) Å, *α* = *β* = *γ* = 90° and **q** = $\tfrac{{\bf{2}}}{{\bf{3}}}$a* + γc*. At 100 K the model presents a pure 4-layer stacking sequence with *γ* = $\tfrac{{\bf{1}}}{{\bf{2}}}$ whereas at the ambient temperature, ordered stacking faults are introduced leading to *γ* < $\tfrac{{\bf{1}}}{{\bf{2}}}$. The model was refined against PEDT data using the dynamical refinement procedure including modulation and twinning as well as against x-ray powder data by the Rietveld refinement.

## Introduction

**Figure 1:**
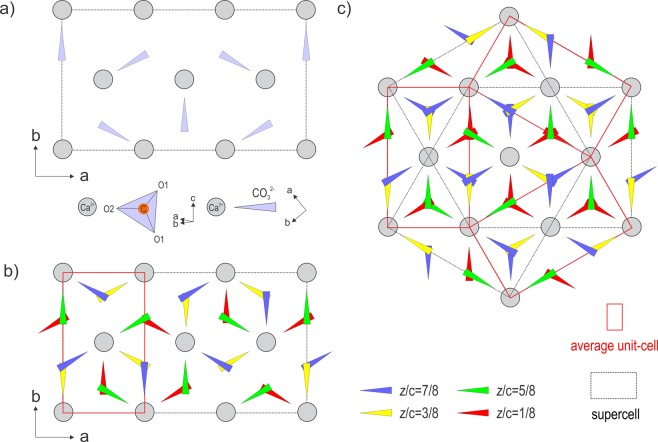
(**a**) Organization of (CO_3_)^2−^ groups within a single layer. (**b**) (CO_3_)^2−^ stacking of the 4-layer polytype. (**c**) Representation of the vaterite structure with the twinning.

Among the three crystallized anhydrous polymorphs of CaCO_3_, vaterite is known to be the least stable form under natural conditions. It has also been identified as a constituent of various biominerals such as crustaceans^1^, statoliths, defective mollusk pearls^2^ and shells^3^, otoliths^4^, ascidians^5^ and even human organic tissues (heart valves for instance^6^) or plants^7^. Vaterite is involved in the first step of crystallization of the two other polymorphs calcite^8^ and aragonite^9^ and in several carbonate forming systems. While its structural determination appears important to understand scaling formation^7^ and biomineralization processes^10^, the exact nature of the vaterite crystal structure has been the subject of ongoing and leaving matters open debates. One Problem arises from the nature of most synthetic and natural forms of vaterite that form nanocrystals not suitable for an x-ray single crystal experiment and limit the solution methodologies. At maximum, the crystallite sizes of vaterite synthesized in controlled conditions do not exceed 60 nm^11^. Another limitation comes from the dual nature of the structure that shows both ordered and disordered features. For these reasons, the early structural studies^12,13^ were carried out on “imperfect” samples showing diffuse streaks, for crystals not larger than 0.1 mm. The various structural models proposed for vaterite are based on unit-cells indexed from powder x-ray^12,14–16^ and neutron diffraction^17^ as well as electron diffraction^18^. An even greater number of theoretically calculated structures have also been proposed^19–24^. The first unit-cell parameter determination of vaterite carried out by Olshausen^14^ resulted in a hexagonal cell with $a=4.11$ Å and $c=8.513$ Å, while the first crystallographic description was done by Meyer in an orthorhombic symmetry^25^. Finally, until the 2000s, the most widely accredited structure was proposed by Kamhi who described a disordered structure^12^. The pseudocell of Kamhi is hexagonal with two layers, space group $P{6}_{3}/mmc$, with ${a}_{0}$ = 4.13 Å, ${c}_{0}$ = 8.49 Å, $Z=2$ and it has CO_3_ groups disordered between three orientations around threefold axes. However, in his original paper Kamhi also reported eight weak diffraction peaks that could not be indexed with his model, resembling a supercell with a lattice rotated by 30° around the $c$ axis and with lattice parameters of $a^{\prime} =\sqrt{3}{a}_{0}$ and $c^{\prime} =2{c}_{0}$. These weak reflections indicate the existence of ordered carbonate groups within a single layer that was also supported later by phase-contrast images^26^. In this way, the organization of the (CO_3_)^2−^ and Ca^2+^ within a single layer is well known (Fig. 1a) according to the steric limitations for carbonate groups to occupy trigonal prisms that are vertically above or next to one another, since that would result in incompatible short distances of 2.04–2.12 Å between oxygen atoms of neighboring carbonate groups. If a single layer is fully ordered, the disorder mainly illustrated by diffuse scattering streaks along the stacking direction must be attributed to variations of the stacking sequence. The single layer was used in monoclinic settings as a basic brick for many of the proposed structural models of vaterite that have been interpreted within the order-disorder (OD) theory which systematizes them as polytypic stackings^27^.

**Figure 2:**
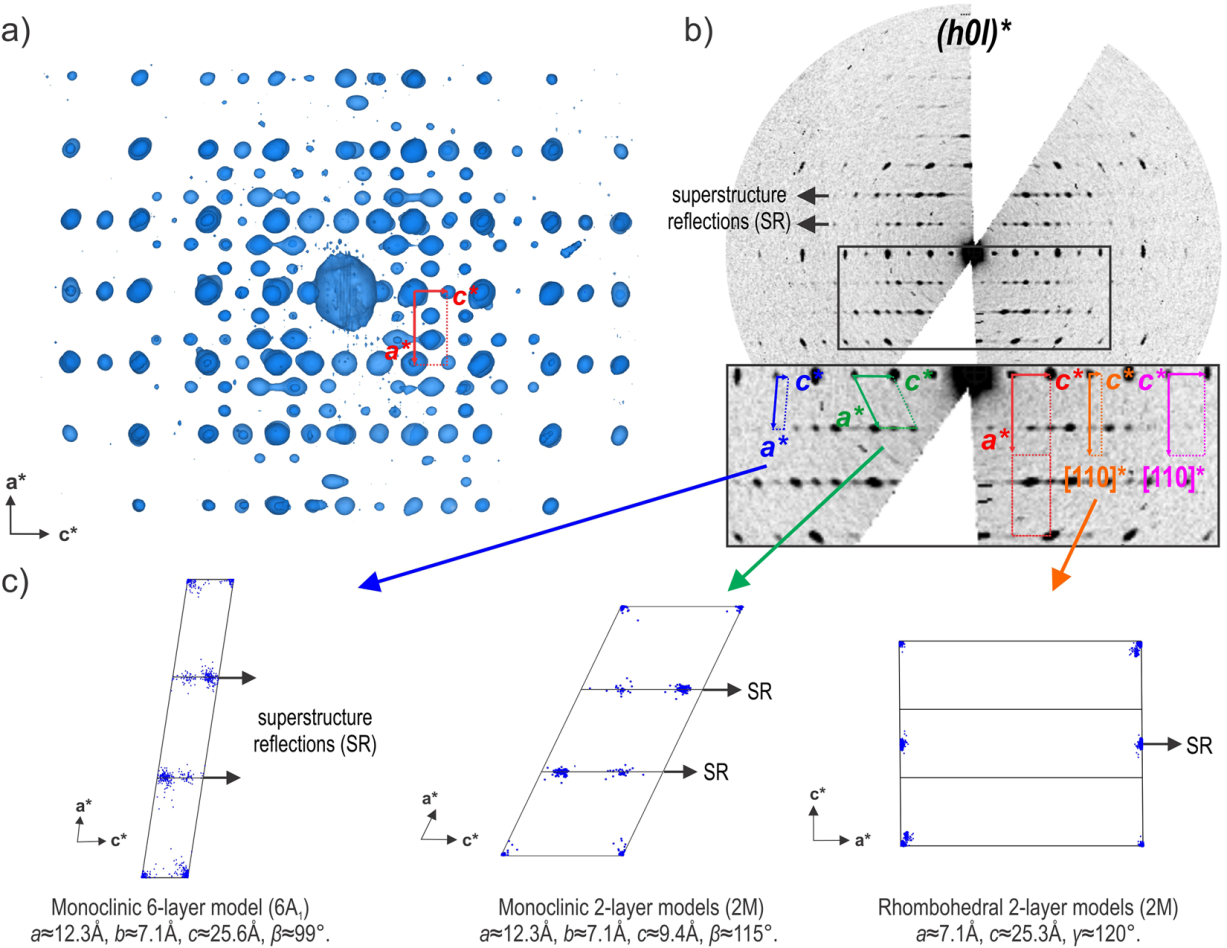
(**a**) Projection along b* of the reciprocal space at 100 K and (**b**) corresponding (*h*0*lm*)* section. On the enlarged (*h*0*lm*)* are represented the indexings of the Kamhi’s hexagonal sub-lattice (pink), the 2 monoclinic lattices of the 6-layer (blue) and 2-layer (green) models, the rhombohedral lattice (orange) and the average unit-cell of the modulated structure (red). (**c**) Corresponding projections of the 3D reciprocal space in 3 unit-cells.

Few years ago, an analysis by Precession Electron Diffraction Tomography (PEDT) was carried out on very small vaterite single crystals^18^. Despite its beam sensitivity, vaterite is a good candidate for electron crystallography. Indeed, at the transmission electron microscope scale, powder consists an assembly of single nano crystals than can be measured and analysed in a similar way as x-ray single crystal data. It was the first time than the vaterite structure was solved ab initio leading to two metrically monoclinic models, one with 6 layers ($C\bar{1}$, $a\sim 12.3$ Å, $b\sim 7.1$ Å, $c\sim 25.6$ Å, $\beta \sim 99^\circ$) and the second with 2 layers ($C2/c$, $a\sim 12.3$ Å, $b\sim 7.1$ Å, $c\sim 9.305$ Å, $\beta \sim 115^\circ$). In their paper, Mugnaioli and co-workers reported the possible modulation along $c$, however the presence of diffuse scattering along the stacking direction for the rows of to the superstructure reflections prevented a precise characterization of the reciprocal space. In Fig. 2, using one of our data set, we show the main indexings used to described the ordered polytypes considered as the most likely according to Christy^28^ as well as the Kamhi’s lattice (Fig. 2b). These three lattices are the monoclinic $a\sim 12.3$ Å, $b\sim 7.1$ Å, $c\sim 9.305$ Å, $\beta \sim 115^\circ$ giving several 2-layer monoclinic models (*C*121^21–24^, *C*2/*c*^18,27^), the monoclinic 6-layer ($C\bar{1}$, $a\sim 12.3$ Å, $b\sim 7.1$ Å, $c\sim 25.6$ Å, $\beta \sim 99^\circ$^18,27^) and the 2-layer rhombohedral lattice ($a\sim 7.1$ Å, $c\sim 25.3$ Å with SG $P{3}_{2}21$^21,23,27^). The reciprocal space is projected in few unit-cells to show the average position of the reflections versus the nodes of the lattices (Fig. 2c). It is obvious that none of these lattices can properly index the superstructure reflections. In the two first cases, they are shifted around 0.16 and 0.33 along the c*-axis from the exact node positions, especially those that are associated later in this paper to the second order satellites in the modulated cell. The rhombohedral lattice does not present the good periodicity along *c*. Because the unit-cells do not index properly the superstructure reflections, the structure solution in these cells leads to an average and not an exact description of vaterite. In the light of new single crystal data collected on more ordered crystals at low temperature by precession electron diffraction tomography (PEDT) with low dose condition, we show that an accurate description of vaterite exists using the superspace formalism to index the superstructure reflections. This superspace appproach allows us to reconcile both ordered and disordered nature of vaterite as well as the evolution observed from low to ambient temperature.

## Results

### Indexing and symmetry analysis

**Figure 3:**
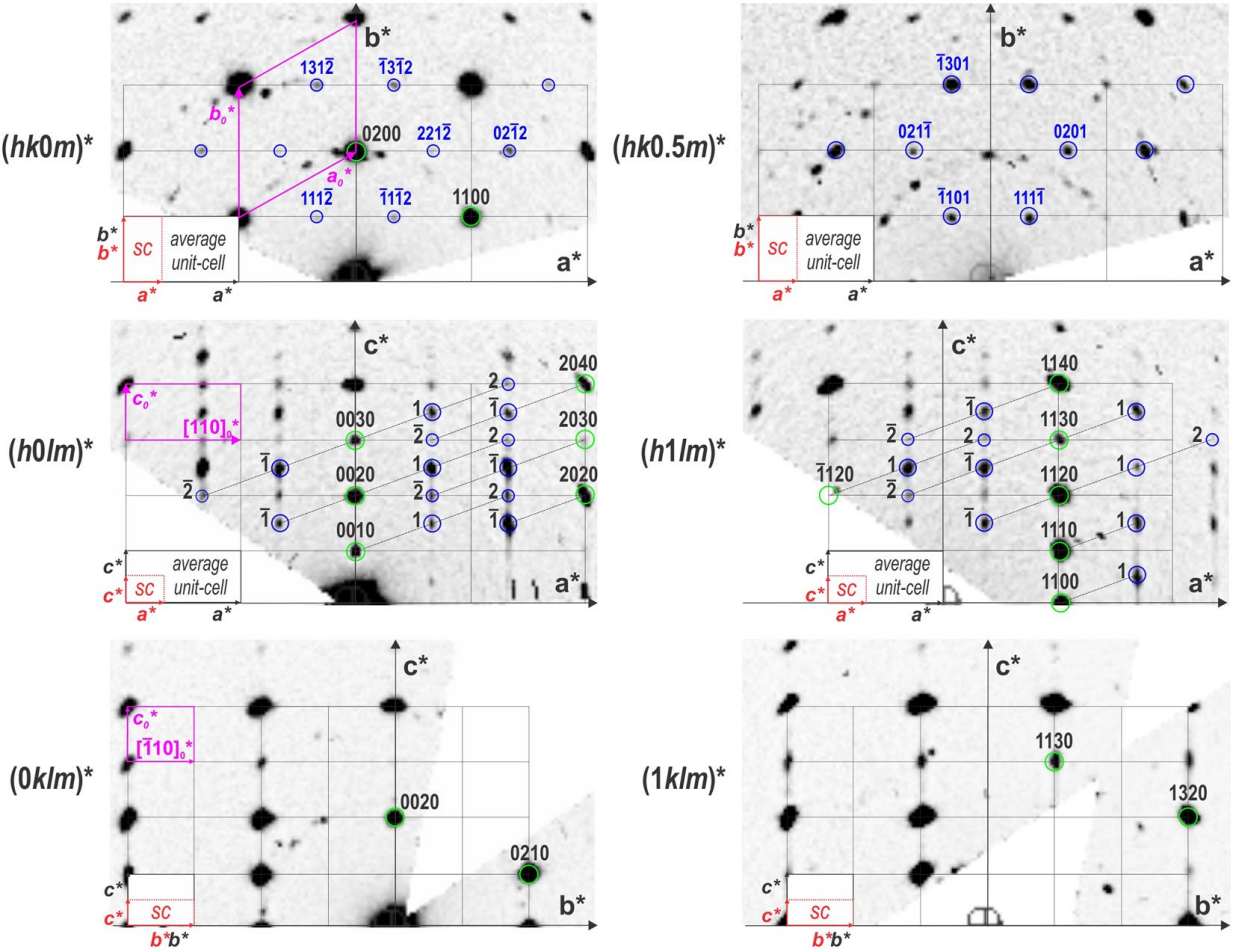
Reciprocal space sections at 100 K with $a\sim 4.1$ Å, $b\sim 7.1$ Å, $c\sim 8.45$ Å, $\alpha =\beta =\gamma =90^\circ$. The picture shows main (green) and satellite (blue) reflections. For comparison the supercell unit-cell (SC) 3*a* × *b* × 2*c* (red) and the hexagonal sublattice (magenta) are given.

**Figure 4:**
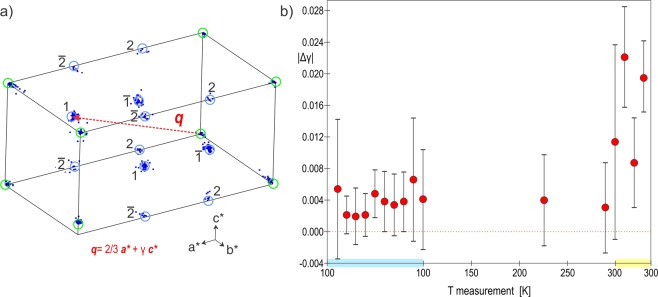
(**a**) Average modulated unit-cell $a\sim 4.1$ Å, $b\sim 7.1$ Å, $c\sim 8.45$ Å, $\alpha =\beta =\gamma =90^\circ$ and **q** = $\frac{2}{3}$a* + *γ*c*, $\gamma \sim \frac{1}{2}$. (**b**) Absolute difference (|Δ*γ*|) between the measured *γ* and the commensurate value $\frac{1}{2}$ for several PEDT data sets following the temperature. The errors displayed correspond to 3*σ*.

**Figure 5:**
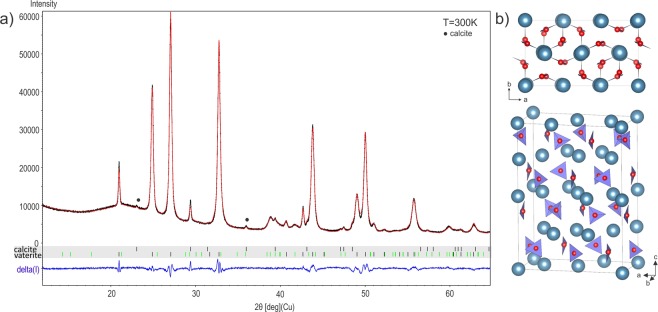
(**a**) XRPD Rietveld refinement diagrams at 300 K in the superspace group $C2$/$c(\alpha 0\gamma )00$ with **q** = $\frac{2}{3}$a* + $\frac{1}{2}$c*. The diagram shows measured (black), calculated (red), and difference (blue) curves. Black and green ticks correspond to main and satellite reflections respectively. (**b**) Representation of the refined model in the supercell 3*a* × *b* × 2*c*.

The sections of the reciprocal space (Fig. 3) exhibit well visible superstructure spots corresponding to an ordered superstructure. While a strong hexagonal pattern with $a=\sqrt{3}{a}_{0}=7.089(9)$ Å $c=2{c}_{0}=16.87$ Å appears in the data, hexagonal or trigonal symmetry cannot lead to an ordered and sterically possible arrangement of the carbonate groups within all the layers. At 100 K the reciprocal space can be indexed using the metrically orthorhombic unit-cell first described by Meyer and used later by Le Bail *et al*.^13,16,25^, $a={a}_{0}=4.086(3)$ Å, $b=\sqrt{3}{a}_{0}=7.089(9)$ Å, $c={c}_{0}=8.439(9)$ Å with an additional commensurate modulation vector ***q*** = $\frac{2}{3}$a* + $\frac{1}{2}$c* up to order 2 to take into account all of the superstructure reflections (Fig. 4a and Table 1). At 100 K, the diffraction data can also be indexed by the 3*a* × *b* × 2*c* supercell, but the global intensity pattern and the extinction conditions are better described using the superspace formalism. At 300 K, similar reciprocal space is observed (Fig. S1 in the Supplementary information) and the same unit-cell is used with a gamma component of the modulation vector showing a small but clear deviation from the commensurate $\gamma =\frac{1}{2}$ for 3 of the 5 data sets (Fig. 4b). At this temperature, crystals are more beam sensitive and disordered, and the reconstructed reciprocal spaces appear less well defined. In order to know if the observed deviation in $\gamma$ is significant, we plotted the absolute difference between the commensurate value 0.5 and the value obtained for each measurement at different temperatures (Fig. 4b). At 100 K none of the measured crystal exhibits real significant deviation from $\gamma =0.5$ and vaterite can be reasonably considered as a commensurate modulated phase or tending to a commensurate case. At 300 K, vaterite becomes incommensurately modulated with a significant distribution in $\gamma$ from crystal to crystal ranging from 0.497(4) to 0.478(2). Because of the incommensurate component along c*, an orthorhombic symmetry is precluded and the symmetry has to be lowered to a monoclinic one. The setting $3{a}_{0}$ × $\sqrt{3}{a}_{0}$ × ${c}_{0}$, $\alpha =\beta =\gamma =90^\circ$ leads to the condition $h+k=2n$ on *hklm* characteristic for a C centering in the supercell as well as in the modulated cell expressed as $(\frac{1}{2},\frac{1}{2},0,0)$. Interestingly, in the section (0*klm*)* the condition $l=2n$ characteristic of a *c* glide plane is present. This apparent glide appears perpendicular to the *a*-axis and is due to the doubling of the *c* parameter. Note that these two symmetry elements are consistent with the predicted near-maximum degree of order 4-layer polytypes *C*2/*c*11 (4M) previously reported^16,21,23,28–30^. However, the modulation vector of the form $\alpha 0\gamma$ requires a *b*-monoclinic superspace group (SSG) setting which cannot account for the c-glide perpendicular to *a*. Instead, a condition $l=2n$ on (*h*0*lm*)* indicates a c-glide in the superspace group perpendicular to *b*. The forbidden reflections $l=2n+1$ visible on (*h*0*lm*)* section of the Fig. 3 can be attributed to residual dynamical scattering or to the presence of an additional 2-layer polytype. This other polytype is present in different proportions in different crystals. In some, the condition related to the c-glide plane perpendicular to *b* is more obvious (see Fig. S2). Further information on the 2-layer polytype is given in the Fig. S3 of Supplementary information and in the Discussion section. All the symmetry considerations lead to the conclusion that a unified description of the vaterite structure is possible in the superspace group (SSG) $C2/c(\alpha 0\gamma )00$. This space group allows a degree of freedom for *γ* that is needed to describe vaterite over all the temperature range. In the supercell 3*a* × *b* × 2*c* this space group becomes $C\bar{1}$. This SSG symmetry and the lattice parameters are consistent with the XRPD diagrams from 300 K to 100 K where all the reflections are indexed except few peaks due to calcite^8^ and aragonite^9^ (Fig. 5). Because the XRPD diagrams from 300 K to 100 K present rather broad peaks of the poorly crystalline and disordered powder samples, the small change in *γ* observed in PEDT experiments is not detectable in the powder XRD diagrams (Fig. S4a). At 100 K, the presence of satellite reflections for half-integer of the Kamhi average 2 layers periodicity along *c* (2 layers ~ 8.49 Å) can already be interpreted as an evidence for the presence of a 4-layer polytype (4 layers ~ 16.98 Å in the supercell). This is supported by previous X-ray^12,16,25,31^ and electron diffraction or HRTEM studies^26,32^. In the literature, several 6-layer polytypes are described and were considered as the best compromise^17,18,21^ but in the modulated setting they would produce satellites at $\gamma =\frac{1}{3}$ that are not observed in our data.

**Table 1:** Lattice parameters obtained from PEDT and XRPD.

**hexagonal subcell from Kamhi (ambient T)** ^12^					
*a*_0_ = 4.13(1) Å	$b={a}_{0}$	*c*_0_ = 8.49(2) Å	$\alpha =\beta =90^\circ \,\gamma =120^\circ$	$V=125.4$ Å^3^	—
**modulated cell from PEDT (100 K)**					
$a\sim {a}_{0}$	$b\sim {b}_{0}\sqrt{3}$	$c\sim {c}_{0}$	$\alpha =\beta =\gamma =90^\circ$	$V=244.38$ Å^3^	$\,\alpha =\frac{2}{3}$,
$a=4.086(3)$ Å	$b=7.089(9)$ Å	$c=8.439(9)$ Å			$\gamma =\frac{1}{2}$
**modulated cell from PEDT (300 K)**					
$a=4.125(9)$ Å	$b=7.140(5)$ Å	$c=8.442(9)$ Å	$\alpha =\beta =\gamma =90^\circ$	$V=248.61$ Å^3^	$\,\alpha =\frac{2}{3}$,
				$$\gamma \in [0.478(2),0.497(4)]$$	
**from XRPD (LeBail) in the modulated cell**					
T[K]	*a*[Å]	*b*[Å]	*c*[Å]	*V*[Å^3^]	d [g.cm^−3^]
100	4.1141(2)	7.1598(3)	8.4634(3)	249.31(2)	2.6665(2)
150	4.1155(2)	7.1636(4)	8.4646(3)	249.55(3)	2.6639(3)
200	4.1189(2)	7.1686(4)	8.4671(3)	250.01(3)	2.6591(3)
250	4.1215(2)	7.1716(4)	8.4683(3)	250.30(1)	2.6559(3)
300	4.1247(1)	7.1761(3)	8.4723(2)	250.772(6)	2.6509(1)

### Ab initio structure determination

**Figure 6:**
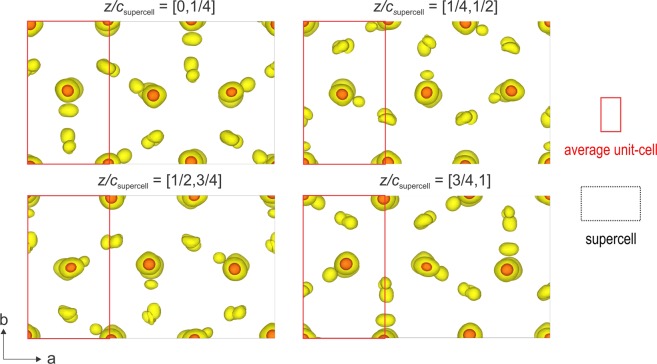
Electrostatic potential map represented layer by layer in the supercell 3*a* × *b* × 2*c*. $z/{c}_{supercell}$ corresponds to ${c}_{supercell}=2c\sim 16.88$ Å.

**Figure 7:**
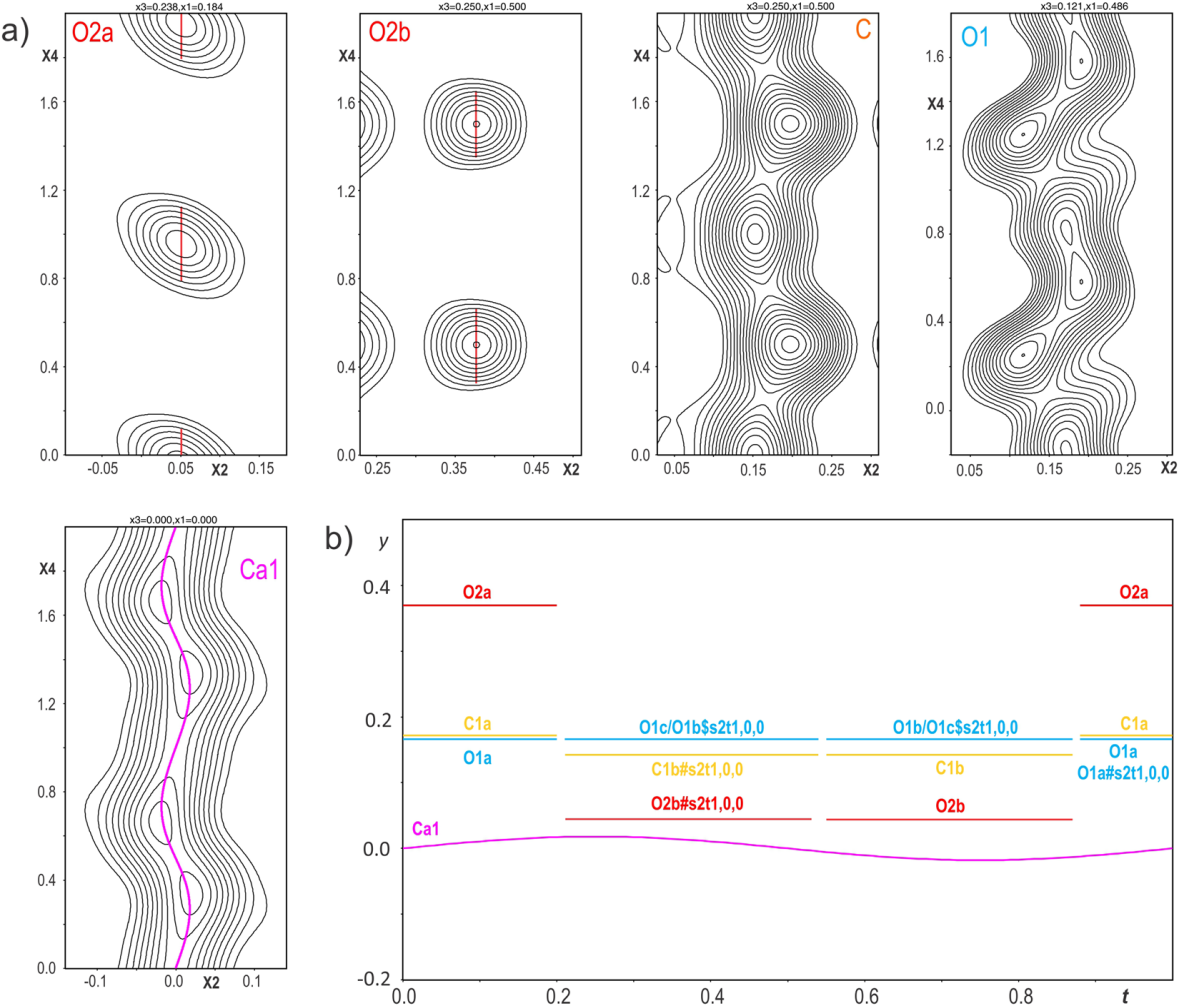
(**a**) *x*2 − *x*4 DeWolf sections calculated around the atoms arising from the charge flipping algorithm. (**b**) *y*/*b* atomic positions following *t* in the modulated unit-cell.

The structure was determined from the combination of 2 PEDT data sets collected at 100 K to increase the data coverage to 93.30% for the resolution of 0.7 Å. The most important experimental parameters are listed in Table S1. The structure solution was performed ab initio with the charge flipping algorithm^33–35^ implemented in the program Superflip^36^ in Jana2006^37^ using the kinematic approximation. A complete structural model with $z=4$ was found in the monoclinic SSG $C12/c1(\alpha 0\gamma )00$ with $\alpha =\frac{2}{3}$ and $\gamma =\frac{1}{2}$. The first solution arising from the charge flipping algorithm is a 3 dimensional map of the electrostatic potential (Fig. 6). To visualize the complete structure and determine the stacking sequence, the map is represented in the supercell 3*a* × *b* × 2*c* layer by layer (the interpreted model is shown in Fig. 1b). As predicted, the carbonate group (CO_3_)^2−^ occupies one half of the Ca_6_ prisms with 3 possible orientations within a single layer (Fig. 1a). To account for this ordered occupancy and orientation, crenel functions are used to define atom sites of the carbonate groups. These crenels are set (${x}_{4}^{0}$: center of the crenel along ${x}_{4}$, with a width Δ) according to the DeWolf sections of the two oxygen sites O2a and O2b that give the orientation of the carbonate (Fig. 7). The third orientation is derived from O2b by symmetry. The carbon and the apical oxygen (denoted O1) sites are split (C1a C1b, O1a O1b and O1c) and also described with crenel functions to correspond to the three possible orientations defined by the O2a and O2b sites. The calcium site remains described with one harmonic function (Fig. 7a). The stacking is defined following the relative orientation of the carbonates from one layer to the next one (+60° = “+”, −60° = “−”, ±180° = “0”) following the notation used by Christy^28^. At 100 K, vaterite is a 4-layer monoclinic polytype (4M) with a stacking expressed as “+0−0”. The same stacking was predicted for a monoclinic 4-layer polytype *C*2/*c*11 by Christy^28^. The predicted symmetry is the highest that can be reached with this (CO_3_) stacking.

### Dynamical refinement

Two refinements were carried out against PEDT data using both “kinematical” and “dynamical” refinements. The results of the kinematical refinements are not shown, since the dynamical refinement provided better results its results are used in the discussion. The refinements had to account for the possible microtwinning already observed (Fig. 1c)^16^. New features added in the program JANA2006 and Dyngo allow us to take into account both modulation and the twinning in the dynamical procedure. Given the strong pseudo-hexagonal lattice geometry, the 3-fold rotations around this axis (±120 degree) were added and improved significantly the results in terms of reliability factors and structural parameters (atomic positions and displacements). The possibility to have the 6-fold twinning is not supported by our data at 300 K because it would produce supplementary satellites that are not observed. The improvement of the *R* factors is more pronounced for the satellite reflections with a decrease around 10 percent. The parameters used to select the reflections involved in the dynamical refinement were chosen following the suggestions in Palatinus *et al*.^38,39^ $R{S}_{g}({\max })=0.4$ at 100 K $R{S}_{g}(max)=0.8$ at 300 K, ${S}_{g}^{(max)}(matrix)=0.01$ Å^−1^, ${g}^{(max)}=1.6$ Å^−1^. At 100 K, one data set was used in the dynamical procedure (crs.2) (Table 2). At 300 K, the crystals are more beam sensitive (incomplete data sets) and present weaker reflection intensities, especially very weak second order satellite reflections. Consequently, two datasets were combined (crs.3, crs.4) and the $R{S}_{g}(max)$ parameter was set to a higher value to include enough reflections in the refinement and ensure a good reflection-to-parameter ratio. Soft restrictions on O-C-O angles were applied to maintain the geometry of the carbonate groups. The second order satellites give much higher *R* factors than the order 1. We assumed that this is because they are very weak and they contain contribution of another polytype. The refinement at 100 K validates the 4-layer model and leads to reliability factors of $R(obs)=15.62 \%$, $wR(obs)=15.79 \%$ (main: $R(obs)=11.84 \%$; order1: $R(obs)=16.98 \%$; order 2: $R(obs)=27.91 \%$). At 300 K, the refinement gives $R(obs)=13.60 \%$, $wR(obs)=13.40 \%$. In order to check if the single crystals measured at the nanoscale are representative of all the sample, a Rietveld refinement from XRPD data at 300 K was performed using the model obtained from the dynamical refinement (see Table S2) and Fig. 5). The Rietveld refinement resulted in ${R}_{p}=2.64 \%$, $w{R}_{p}=3.76 \%$, ${R}_{B}=3.03 \%$ and $R(obs)=1.98 \%$ and with a density $d=2.6509(1){\rm{g}}.{\mathrm{cm}}^{-3}$ similar to the Kamhi’s one.

**Table 2:** Dynamical refinements from PEDT data in the SSG $C12$/$c1(\alpha 0\gamma )00$.

**100** **K**								
**atomic positions**								
***atoms***	***Occ***.		***x/a***	***y/b***	***z/c***	***Uiso*** **[Å** ^**3**^ **]**	${{\boldsymbol{x}}}_{{\bf{4}}}^{{\bf{0}}}$	**Δ**
Ca1	0.5		0	0	0	0.0151(6)	—	—
		*sin*	0.0317(7)	−0.0147(4)	−0.0050(9)			
C1a	0.33		0	0.2962(8)	0.25	0.0077(14)	0.5	1/3
C1b	0.33		−0.0565(13)	0.3599(10)	0.2254(6)	0.0077(14)	0.1167(9)	1/3
O1a	0.33		0.0134(17)	0.3856(8)	0.1199(6)	0.0056(8)	0.4439(12)	1/3
O1b	0.33		−0.0975(13)	0.3214(10)	0.1481(9)	0.0056(8)	0.7173(19)	1/3
O1c	0.33		0.0823(13)	0.3431(8)	0.0964(9)	0.0056(8)	1.1447(19)	1/3
O2a	0.33		0	0.1102(12)	0.25	0.0099(11)	0.50	1/3
O2b	0.33		−0.3389(15)	0.4351(10)	0.2327(9)	0.0099(11)	−0.0679(11)	1/3
**refinement parameters**								
	***N***(***obs***)	***N***(***all***)	***R***(***obs***)	***wR***(***obs***)	***R***(***all***)	***wR***(***all***)	***N*** ***par***.	***t***(**2**)**[Å]**
all	2170	4395	15.62	15.79	24.57	16.24	122	288(5)
main	696	939	11.84	13.94	15.28	14.13		
sat.1	1007	1738	16.98	16.58	25.79	17.17		
sat.2	478	1756	27.91	33.19	45.41	34.00		
twin fractions crs.2: fract.1 = 0.377(11), fract.2(+120°) = 0.308(8), fract.3(−120°) = 0.315(8)								
**300** **K**								
**atomic positions**								
***atoms***	***Occ***.		***x/a***	***y/b***	***z/c***	***Uiso*** **[Å** ^**3**^ **]**	${{\boldsymbol{x}}}_{{\bf{4}}}^{{\bf{0}}}$	**Δ**
Ca1	0.5		0	0	0	0.0186(4)	—	—
		*sin*	0.0363(9)	−0.0135(4)	−0.0009(6)			
C1a	0.33		0	0.2978(9)	0.25	0.0049(9)	0.5	1/3
C1b	0.33		−0.0838(14)	0.3453(12)	0.2371(3)	0.0049(9)	0.1046(9)	1/3
O1a	0.33		−0.029(2)	0.3886(9)	0.1175(3)	0.0262(7)	0.417(2)	1/3
O1b	0.33		−0.0862(15)	0.3213(15)	0.1328(4)	0.0262(7)	0.720(3)	1/3
O1c	0.33		0.0544(16)	0.3203(14)	0.1014(4)	0.0262(7)	1.132(3)	1/3
O2a	0.33		0	0.1220(11)	0.25	0.0310(11)	0.50	1/3
O2b	0.33		−0.3593(14)	0.4141(11)	0.2441(11)	0.0310(11)	−0.0757(10)	1/3
**refinement parameters**								
	***N***(***obs***)	***N***(***all***)	***R***(***obs***)	***wR***(***obs***)	***R***(***all***)	***wR***(***all***)	***N*** ***par***.	***t***(**3**)**[Å]**
all	1530	9789	13.60	13.40	42.81	15.04	139	121(2)
main (3)	381	908	9.37	9.89	18.48	10.30		
sat.1 (3)	325	1692	14.64	15.27	45.78	14.44		
sat.2 (3)	101	1864	28.84	33.88	75.58	44.07		*t*(4)[Å]
main (4)	338	869	11.53	11.32	22.67	13.42		142(2)
sat.1 (4)	291	1966	22.87	21.77	61.25	24.82		
sat.2 (4)	59	1976	30.44	42.09	84.91	51.69		
twin fractions crs.3: fract.1 = 0.31(1), fract.2 = 0.332(7), fract.3 = 0.326(9)								
twin fractions crs.4: fract.1 = 0.301(12), fract.2 = 0.343(8), fract.3 = 0.326(10)								

## Discussion

**Figure 8:**
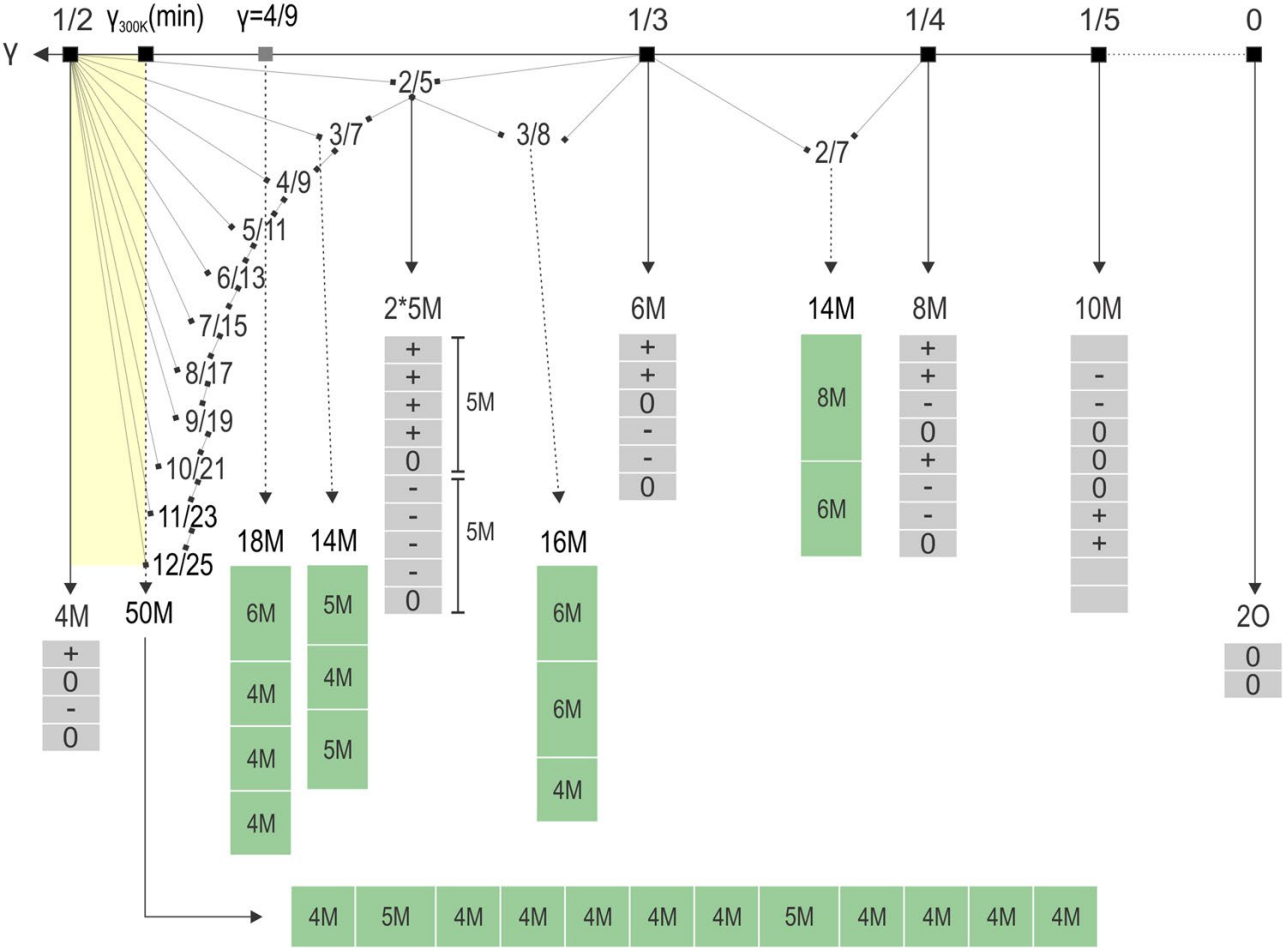
Considering an theoretical incommensurate modulation along c* from $\gamma =0$ to $\gamma =\frac{1}{2}$, the possible stackings can be described following a Farey tree hierarchy^40^ using the stacking of the basic bricks (2*5M, 6M, 8M, etc...) and the two “end members” 4M and 2O. The possible macroscopic ordered sequences observed at 300 K are within the yellow range of *γ*.

A smooth transition is observed from 100 K to 300 K from a commensurate (or quasi commensurate) to an incommensurate modulated phase. In the PEDT data, the main reflections are not affected by the diffuse scattering showing that the calcium hexagonal substructure is maintained and the disorder is mostly related to stacking faults of the carbonate groups along *c*. despite the presence of diffuse features, the ED pattern remains mainly ordered showing there is a predominant long range order in the stacking sequence. According to our data at 300 K, macroscopic vaterite polytypes exist for $\gamma$ ranging from $0.478(2)\sim \frac{12}{25}$ to $\frac{1}{2}$ (yellow area in Fig. 8). When $\gamma$ goes below $\frac{1}{2}$ at higher temperature, ordered stacking faults are introduced in the pure 4-layer stacking giving a global stacking sequence with a majority of 4 layers (4M) with some blocks made of 5 (5M) layers and sometimes 6 layers (6M). A way to visualize the evolution of the stacking sequence along *c* depending on *γ* is to use a Farey tree hierarchy^40^. This tree can be split in two parts, the part associated to possible ordered long range stacking from ${\gamma }_{300K}({\min })\sim \frac{12}{25}$ to $\frac{1}{2}$ and the part related to the disorder. In this way, the Farey tree hierarchy can also be used as a trick to explain the diffuse features due to more local stacking sequences that appear as domains by setting any $\gamma$ value along those diffuse stripes assuming a possible Bragg peak for each specific value. Thereby, a corresponding stacking sequence appearing only localy can be determined as well as the type of stacking fault needed to disturb the perfect 4-layer sequence. On such Farey hierarchy (Fig. 8), the end members are represented by the 4-layer (4M) and the 2-layer (2O) polytypes corresponding to $\gamma =\frac{1}{2}$ and 0 respectively. All theoretical sequences represented in the Fig. 8 are obtained from the ab initio procedure in the Superflip program using the data of the 4-layer polytype and attributing the intensities of the satellite reflections to another $\gamma$ value. The same stacking sequences can be built up from the model initially designed for the 4M polytype by adjusting $\gamma$ to another value. In the literature two stacking sequences were suggested as potential 2-layer polytype: “00” (2O: *Cmcm*) and “+−” (2M: *C*12/*c*1). The later 2M is used as the basic brick in the description using the OD theory by Makovicky^27^. However, using one of the two methods to predict the sequence using our unified modulated model, the derived stacking of the 2-layer end member came out to be “00”. For specific $\gamma$ values like $\frac{1}{3}$, $\frac{1}{4}$ or $\frac{2}{5}$, the stacking sequences are not a combination of other sequences and are also used as basic bricks together with the 4M or 2O to obtain all the other sequences. For example, when $\gamma =\frac{4}{9}$, the characteristic stacking sequence is a combination of 3*4M + 1*6M = 18M. Indeed $\frac{4}{9}$ can be decomposed as $\frac{1+1+1+1}{2+2+2+3}$ by adding both numerators and denominators. This value is not observed in our data as a sharp satellite but it would correspond to the theoretical position of the reflections of the 6-layer model from Mugnaioli and co-workers and may appear locally ($\,C\bar{1}$, $\,a\sim 12.3$ Å, $b\sim 7.1$ Å, $c\sim 25.6$ Å, $\beta \sim 99^\circ$)^18^. Another example is the theoretical rhombohedral 2-layer model $P{3}_{2}21$ considered by Demichelis *et al*. as one of the most stable possible polytype^21^. It would produce reflections for $\gamma =\frac{1}{3}$ that correspond in the tree to a pure 6-layer sequence “+ + 0 − − 0”. The transition mechanism must involve flips of the carbonate groups within the Ca_6_ cavities around the *c*-axis. The possible CO_3_ rotation is already known for the aragonite-calcite transition and is also associated to disorder^41^. We cannot exclude that the temperature dependent transition is related with beam damage, faster at room temperature.

A recurring issue in the understanding of vaterite concerns the possible coexistence of several polytypes. Different studies by Solid State Nuclear Magnetic Resonance (SSNMR), quantum mechanical ab initio calculations based on Density Functional Theory studies carried out on different orthorhombic, hexagonal and monoclinic models conclude the existence of several polytypes explains their data^21,24^. Another work by Kabalah-Amitai and co-workers^26^ characterized vaterite from spicules of *H*. *momus* ascidians from HRTEM analysis^26^. The main structure is proposed to exhibit hexagonal symmetry consistent with the original substructure reported by Kahmi, whereas a minor structure with unknown structural symmetry and *d*-spacing ~ 3.63(5) Å is suggested to explain the additional lattice fringes in the HRTEM images^26^. This *d*-spacing is not found in our modulated model showing that this additional phase is not responsible for the superstructure reflections. An unknown impurity characterized by only one broad peak in the XRPD diagram of the sample A corresponds to this *d*-spacing ~ = 3.66 Å (Fig. S4b). However, this phase was never found as a single crystal and measured by PEDT and is probably highly disordered. Considering that all the reflections are indexed with our 4M model, no other polytype is needed to explain our data. The only possible additional ordered polytype is a coherent 2-layer intergrowth $a=3{a}_{0}\sim 12.38$ Å, $b\sim {a}_{0}\sqrt{3}$ Å, $c={c}_{0}\sim 8.44$ Å that produces reflections superimposed to the average lattice and the second order satellites (Fig. S3). This polytype corresponds to the 2-layer end member for $\gamma =0$ with a stacking “00” (2O).

Looking closer at the structural model and within the precision that can be reached by the refinement, the vaterite model remains very similar from 100 K to 300 K. The evolution with temperature is mainly accompanied by crystal faulting and disorder. Despite one symmetry independent Ca atomic site, the modulation produces 2 types of Ca coordinations (Fig. S5). All the distances C-O1 are found between 1.239(9) Å and 1.303(5) Å and C-O2 1.241(9) Å and 1.325(10) Å. The O-O distances between 2.171(10) Å and 2.251(9) Å (Table 3). Our structural determination interestingly shows some deviations from Kamhi’s model of vaterite. The C-O distances by Kamhi range from 1.22(11) Å to 1.28(4) Å, while we observe deviations towards larger (1.325(10) Å for C1a-O2a) values. This is a sign of the larger instability of this phase resulting in its “metastability” compared to calcite and aragonite. One interesting feature concerns the aplanarity of the carbonate groups^42^. Pokroy^42^ first established in biogenic aragonite the slight shift of C atoms out of the oxygen planes, a shift around 0.05 Å compared to their geological reference, and varying from mollusc species to species. Data by Caspi *et al*. indicated a value closer to 0.04 Å^43^ (Table 3). However, Chateigner *et al*. questioned the possibility of accurately determining such small quantities due to the averaging of powder diffraction data^44^. In vaterite, the aplanarity of about 0.055 Å supports the instability theory. The *δ* values giving rise to ~1 kJ.mol^−1^ of energy difference between calcite (*δ* = 0 Å) and aragonite (*δ* = 0.042(1) Å), reaches ~0.055 Å in vaterite at 300 K, explaining its fairly large instability, and its potential (and observed in practice) destabilisation into one of the two or both former phases, depending on the conditions. Even larger *δ* values were already observed in some layers of gastropod shells^44^. The aplanarity in aragonite is usually believed to come from mineral-macromolecule interactions, which also gives rise to unit-cell distortions. But since no macromolecule was added to the solution, the only cause of larger *δ* stabilization is purely mineral. We conclude that relatively large values of *δ* in calcium carbonate can be stabilized by purely mineral processes, including defect incorporation eventually and not necessarily under biogenic molecular action.

**Table 3:** Comparison of C-O distances, O-C-O angles and the aplanarity for the three calcium carbonate polymorphs with vaterite described in our refined modulated model as well as the Kamhi’s model.

Vaterite		Calcite	Aragonite	Kamhi
100 K	300 K	298 K	298 K	298 K
**C-O distances [Å]**				
C1a-O1a: 1.274(6)	C1a-O1a: 1.303(5)	1.284(2)	C-O1: 1.288(2)	C-O1: 1.22(11)
C1a-O2a: 1.325(10)	C1a-O2a: 1.261(10)		C-O2: 1.2835(13)	C-O2: 1.28(4)
C1b-O1b: 1.274(9)	C1b-O1b: 1.317(6)			
C1b-O1c: 1.239(9)	C1b-O1c: 1.296(6)			
C1b-O2b: 1.278(9)	C1b-O2b: 1.241(9)			
**O-C-O angles [degree]**				
O1a-C1a-O1a: 120.0(6)	O1a-C1a-O1a: 120.0(6)	120	O1-C-O2: 120.11(7)	O1-C-O2: 120(3)
O1a-C1a-O2a: 120.0(3)	O1a-C1a-O2a: 120.0(3)		O2-C-O2: 119.64(13)	O2-C-O2: 119(4)
O1b-C1b-O1c: 119.6(6)	O1b-C1b-O1c: 119.4(6)			
O1b-C1b-O2b: 120.0(6)	O1b-C1b-O2b: 120.0(5)			
O1c-C1b-O2b: 120.0(6)	O1c-C1b-O2b: 120.0(5)			
**aplanarity** ***δ*** **[Å]**				
*δ*(C1a) = 0	*δ*(C1a) = 0	*δ*(C) = 0	*δ*(C) = 0.0266(17)	—
*δ*(C1b) = 0.050(7)	*δ*(C1b) = 0.0603(88)			—

## Conclusion

Our results show that vaterite grows mostly as an ordered monoclinic 4-layer polytype. Its structure at 100 K was solved ab initio and described using the superspace formalism as commensurately modulated structure with the superspace group $C12$/$c1(\alpha 0\gamma )00$ with $\alpha =\frac{2}{3}$ and $\gamma =\frac{1}{2}$. The model was refined against electron diffraction data using dynamical approach including both modulation and twinning as well as a Rietveld refinement against XRPD data. Similarly to what is observed in aragonite, the (CO_3_) aplanarity of about 0.055 Å present in vaterite explains its metastability. At temperatures around 300 K, the structure stabilizes by introducing ordered stacking faults responsible for the incommensurability in the stacking direction giving rise to some 5-layer or 6-layer blocks among the average 4-layer stacking sequence. At this stage, there still remain some unanswered questions concerning vaterite. Since the dynamical refinement does not take into account of the disordered features, a better understanding of the occurrence of the disorder and twinning, especially at the ambient temperature, would require a quantitative analysis of the diffuse scattering. In the same way, the results are obtained on synthetic samples and we cannot exclude possible variations in natural biotic and abiotic vaterite.

## Methods

Two vaterite samples were synthesised by precipitations from solutions in doubly distilled water. Calcium chloride CaCl_2_, sodium Na_2_CO_3_ and potassium K_2_CO_3_ carbonates from Prolabo were used as reactants. The crystallisation of the first sample (A) was obtained by mixing two 0.1M equimolar solutions of CaCl_2_ and Na_2_CO_3_ at 25 °C, with the solution maintained at this fixed temperature under magnetic stirring for 15 min. The second sample (B) was obtained using CaCl_2_ (3M) and K_2_CO_3_ (1M) solutions mixed in 50 mL and 450 mL water respectively. Both solutions were preheated at 33 °C, mixed in a beaker and stirred for 20 min at the same temperature. The precipitates obtained in the two preparations after filtration were washed actively with water and methanol, and then were dried in a desiccator under vacuum for 48 h, resulting in finely grained powders.

For transmission electron microscopy (TEM) investigations, the white powder was slightly dispersed in ethanol and a drop of the suspension was deposited and dried on a copper grid with a thin film of holey amorphous carbon. Electron diffraction investigations were performed on a Philips CM120 electron transmission microscope (TEM) (Vacc = 120 kV, LaB_6_) with the precession device Nanomegas Digistar and a side-mounted CCD camera Olympus Veleta with 14 bit dynamic range. PEDT data sets of non-oriented patterns were recorded at 100 K, 225 K, 290 K and 300 K on several thin crystals or clusters of crystals (Fig. S6). The precession angle was set to 1 degree with a tilt step of 1 degree. A condenser aperture of 10 *μ*m and low illumination setting (spot size 8) were used to produce a semi-parallel beam of 60–85 nm in diameter on the sample to reduce the electron dose. Fifteen data sets were analysed to determine average lattice parameters and symmetry of the crystals but only two data sets collected at 100 K (crs.1 and crs.2) and two at 300 K (crs.3 and crs.4) were used for the structure solution and refinement (see experimental details in Table S1). PEDT data were analyzed using the computer programs PETS^45^ and JANA2006^37^ following a procedure described elsewhere^46^. For each data set the result is a list of *hklm* indices with associated intensities and estimated standard deviations based on counting statistics. The refinements were performed using both kinematical and dynamical approaches (“kinematical refinement” and “dynamical refinement”) implemented in JANA2006^38,39^.

X-ray powder diffraction (XRPD) diagrams were measured from 300 K to 100 K with 50 K steps. The pulverized sample B was placed in the 0.5 mm diameter borosilicate glass capillary and measured in the transmission Debye-Scherrer configuration on the SmartLab diffractometer of Rigaku, which was equipped with a Cu rotating anode at 9 kW, primary Johansson monochromator combined with focusing mirror and detector D/tex Ultra 250. The temperature of the sample was controlled using a Cryostream 700 cooling system. Powder patterns were measured from 12° to 65° 2*θ* with a step size of 0.015° 2*θ* and with a speed of approx. 0.1°/min. The sample A was measured in the same conditions at 100 K.

## Supplementary information

Supplementary information : Stacking sequence variations in vaterite resolved by precession electron diffraction tomography using a unifed superspace model
