# Supplementary material for: Stacking sequence variations in vaterite resolved by precession electron diffraction tomography using a unified superspace model
Source: Sci Rep. 2019 Jun 24;9:9156. doi: 10.1038/s41598-019-45581-6 (PMC6591425; doi:10.1038/s41598-019-45581-6)

Supplementary information : Stacking sequence variations in vaterite resolved by precession electron diffraction tomography using a unified superspace model

Gwladys Steciuk[1,*], Lukáš Palatinus[1], Jan Rohlicek[1], Salim Ouhenia[2], and Daniel Chateigner[3,**]

[1]Institute of Physics of the Czech Academy of Sciences, Na Slovance 2, Prague, Czech Republic.
[2]Laboratoire de Physique, Faculté des Sciences et Sciences de l'ingénieur, Béjaïa, 06200, Algeria.
[3]CRISMAT, Normandie Université, ENSICAEN, UNICAEN, CNRS UMR6508, 6 Bd Maréchal Juin, F-14050 Caen Cedex 4, France.
[*]steciuk@fzu.cz
[**]daniel.chateigner@ensicaen.fr

	crs.1	crs.2	crs.3	crs.4
sample	A	A	B	B
T measurement [K]	100	100	300	300
angular range [deg]	91	101	63	80
$sin\theta/\lambda$ [Å^{-1}]	0.7	0.7	0.708	0.7
Coverage	63.71%	71.56%	50.09%	83.45%
N(obs/all)indep.(kin)	2211/3591	2345/3969	366/1327	349/1652
$Rint$(obs/all)(kin)	19.91/20.77	17.69/18.71	13.44/19.84	11.02/18.25
Redundancy	3.226	3.155	1.911	1.926
N(obs/all)(dyn)	10272/21077	11180/23417	1923/14338	1895/18202
data combination at 100K (crs.1 + crs.2) 2 order satellites				
N(obs/all) indep.(kin)	$Rint$(obs/all)	coverage	$sin\theta/\lambda$	redundancy
1764/2688	23.92/24.96	93.30%	0.708 Å $^{-1}$	2.935
data combination at 300K (crs.3 +crs.4) with 2 order satellites.				
N(obs/all) indep.(kin)	$Rint$(obs/all)	coverage	$sin\theta/\lambda$	redundancy
541/2046	31.08/38.16	84.61%	0.708 Å $^{-1}$	2.794

Table S1: PEDT experimental details. The statistics are evaluated in the superspace group $C12/c1(\alpha0\gamma)00$ and the reflections are observed for I¿$3\sigma(I)$.

**atomic positions**								
atoms	*Occ.*		x/a	y/b	z/c	$Uiso$[Å^3]	x_4^0	Δ
Ca1	0.5		0	0	0	0.0284(18)	-	-
		sin	0.073(2)	-0.0185(11)	0			
C1a	0.33		0	0.328(5)	0.25	0.0055(18)	0.5	1/3
C1b	0.33		-0.089(4)	0.345(3)	0.240(3)	0.0055(18)	0.1026	1/3
O1a	0.33		0.020(5)	0.426(3)	0.118(2)	0.0055(18)	0.448(4)	1/3
O1b	0.33		-0.022(4)	0.311(3)	0.1376(11)	0.0055(18)	0.762(6)	1/3
O1c	0.33		0.080(4)	0.288(2)	0.095(2)	0.0055(18)	1.143(6)	1/3
O2a	0.33		0	0.150(3)	0.25	0.0055(18)	0.50	1/3
O2b	0.33		-0.398(3)	0.4064(14)	0.221(2)	0.0055(18)	-0.113(2)	1/3
**refinement parameters**								
	$N(obs)$	$N(all)$	$R(obs)$	$wR(obs)$	$R(all)$	$wR(all)$	$Npar.$	$fract1.$
all	428	436	1.98	3.71	1.98	3.71	20	98.22(5)%
main	79	85	2.28	5.76	2.28	5.76		
sat.1	174	174	1.60	2.96	1.60	2.96		
sat.2	175	177	2.03	3.37	2.04	3.37		
**profil R factors:**								
GoF=3.20%,	R_p=2.64%,	wR_p=3.76%,	$R_B(obs)$=3.03%					

Table S2: XRPD Rietveld refinement at 300K of vaterite in SSG $C12/c1(\alpha0\gamma)00$.

Figure S1: Reciprocal space sections at 300K in the unit-cell a ∼4.1 Å, b ∼7.1 Å, c ∼8.45 Å, $\alpha = \beta = \gamma = 90°$. Some of the main (green) and satellite (blue) reflections as well as the corresponding supercell (SC) $3a$ x b x $2c$ are indicated.

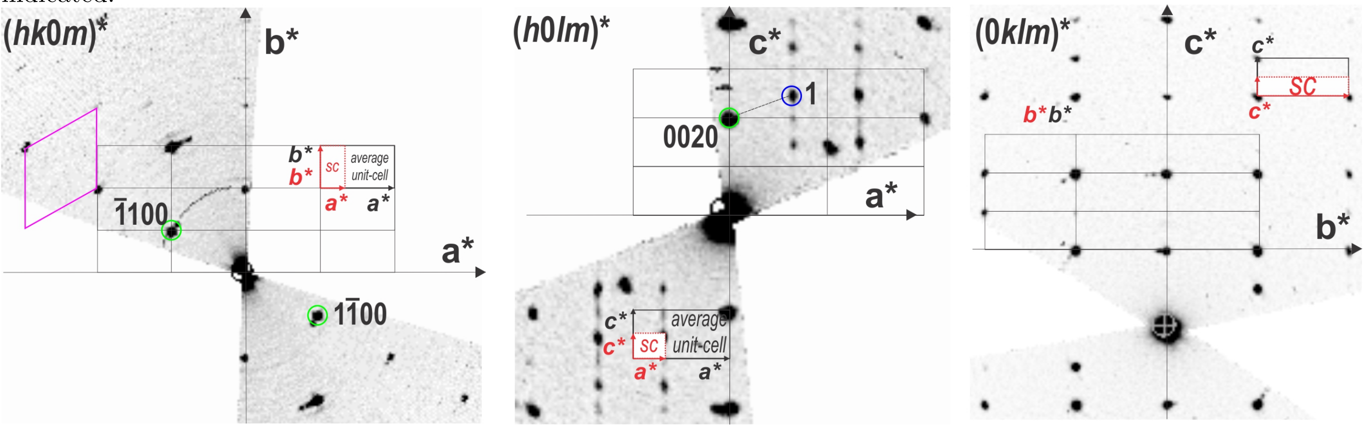

Figure S2: Sections at 100K for another vaterite crystal presenting more obvious $h = 2n$ condition on $(h0lm)^*$ related to the c glide plane perpendicular to b.

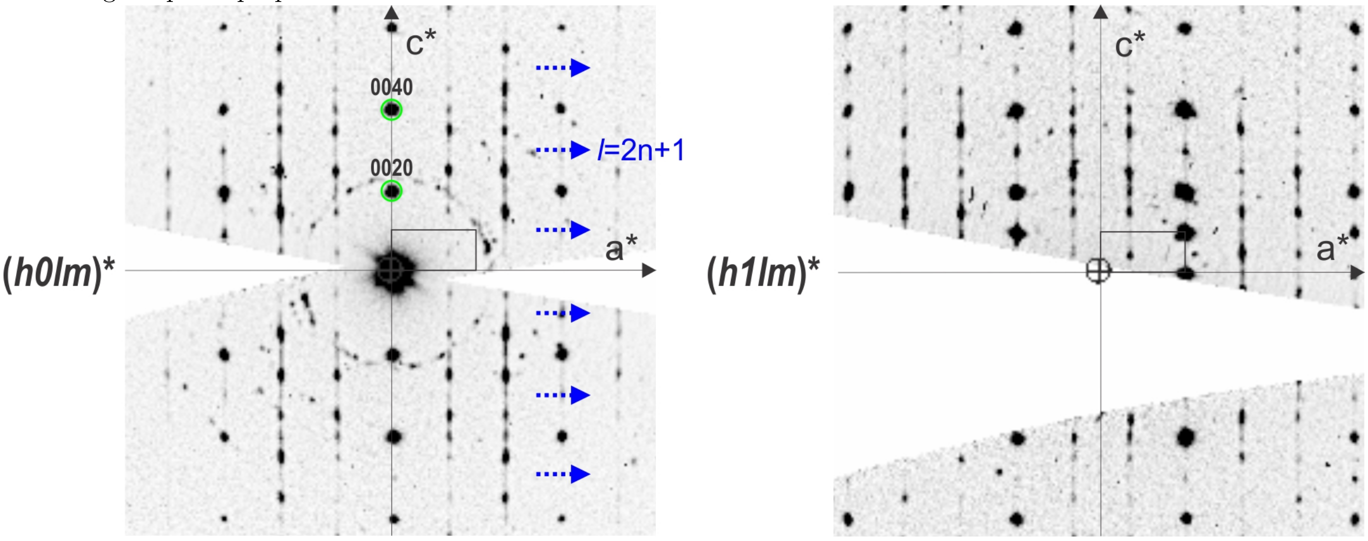

Figure S3: Two theoretical $(h0lm)^*$ sections are represented. They correspond to a) the 4-layer (4M) polytype and b) the 2-layer polytype at 100K with their respective models. If the 2-layer polytype is present as a coherent intergrowth with the 4M one, all the reflections will be superimposed except for the reflections $l = 2n + 1$ that come only from the second polytype (or the residual dynamical effects). We assumed that the reflections $l = 2n + 1$ are not only due to residual dynamical scattering because when they are weak in some data set, they are associated to weaker second order satellites.

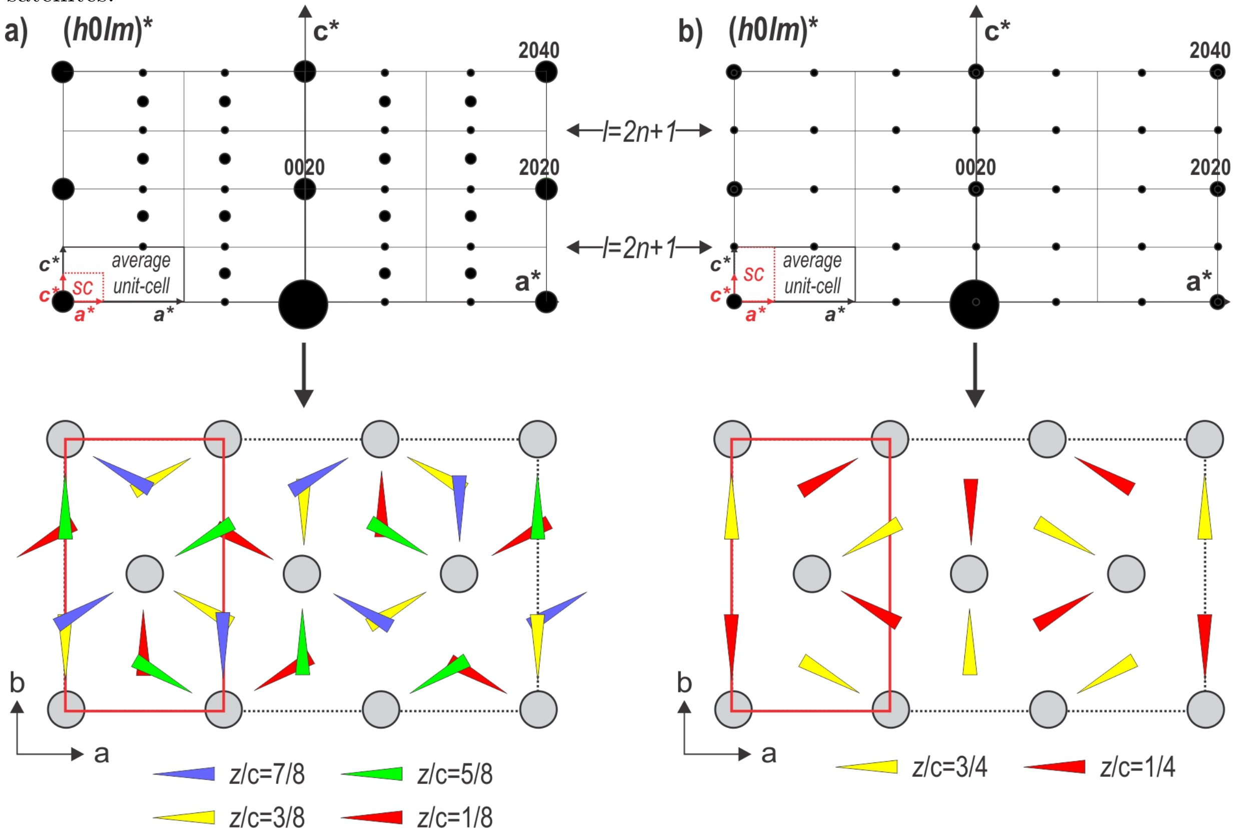

Figure S4: a) XRPD diagrams of vaterite from 100K to 300K collected on the sample B. b) XRPD diagram at 100K of the sample A.

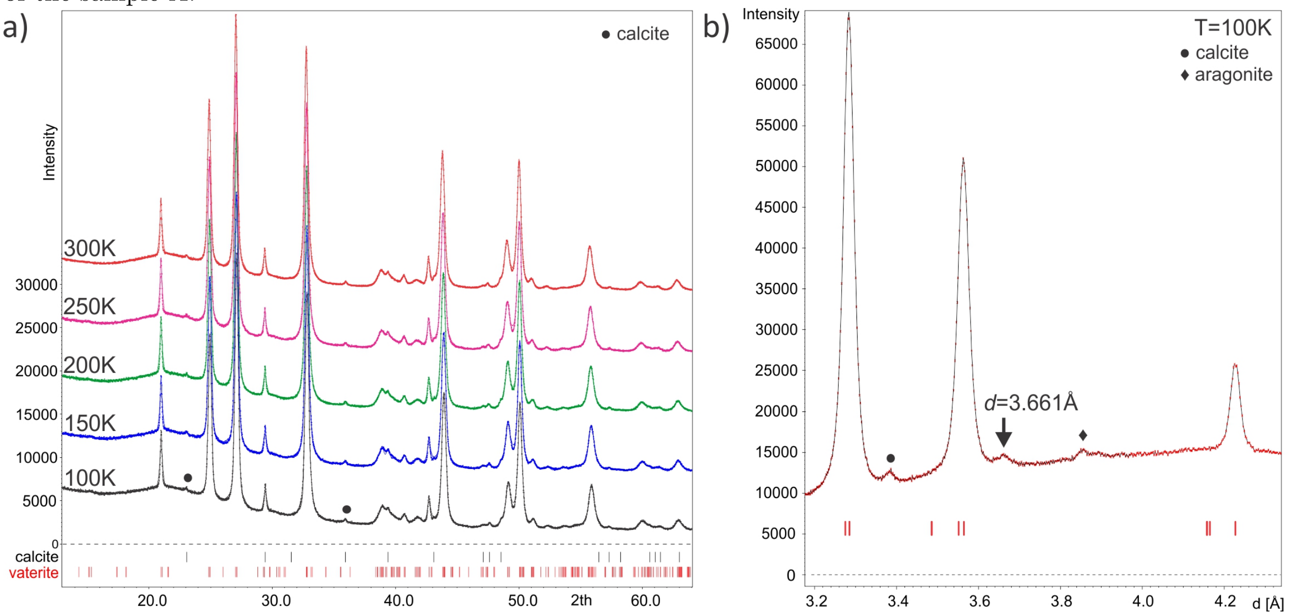

Figure S5: a) Ca-O distances with t and b) the two Ca environments produced by the modulation of Ca atomic site.

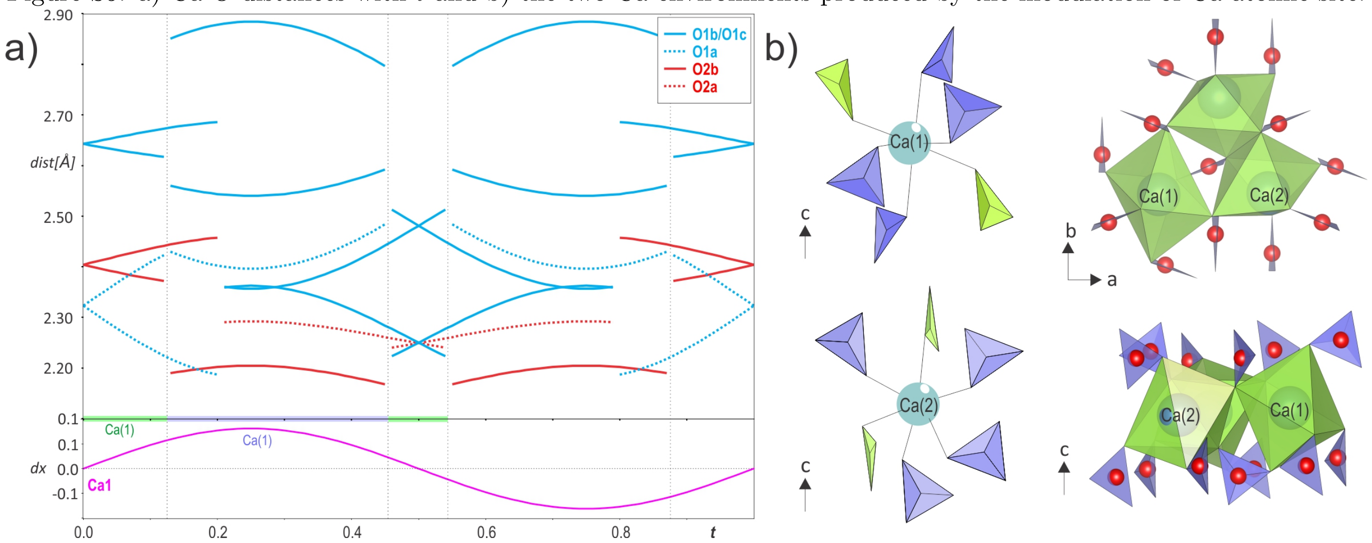

Figure S6: Sample morphology and typical beam size used for the PEDT collections.

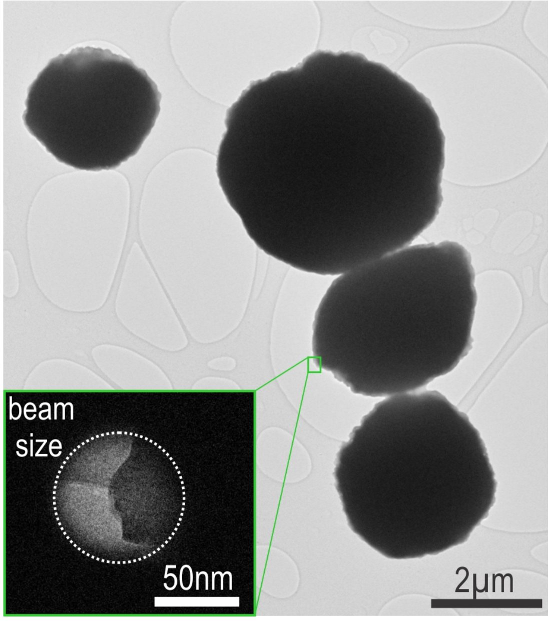

Supplement: Supplementary file 1 — Supplementary information : Stacking sequence variations in vaterite resolved by precession electron diffraction tomography using a unifed superspace model [file 41598_2019_45581_MOESM1_ESM.pdf]
